# Lead detour

**DOI:** 10.1007/s12471-019-01320-0

**Published:** 2019-08-12

**Authors:** L. M. van den Broek, S. W. Westra, R. Evertz, M. Boulaksil

**Affiliations:** Department of Cardiology, Radboud University Medical Centre, Nijmegen, The Netherlands

A 48-year-old male was brought to our Emergency Department after a car accident due to syncope. His medical history included paroxysmal atrial fibrillation and a bicuspid aortic valve requiring aortic valve replacement with a mechanical prosthesis. At presentation, the electrocardiogram showed a new third-degree atrioventricular block. It was decided to implant a dual-chamber pacemaker, but the procedure was more challenging than expected. Fig. 1 shows the position of the leads at the end of the procedure. What is your diagnosis?

**Fig. 1:**
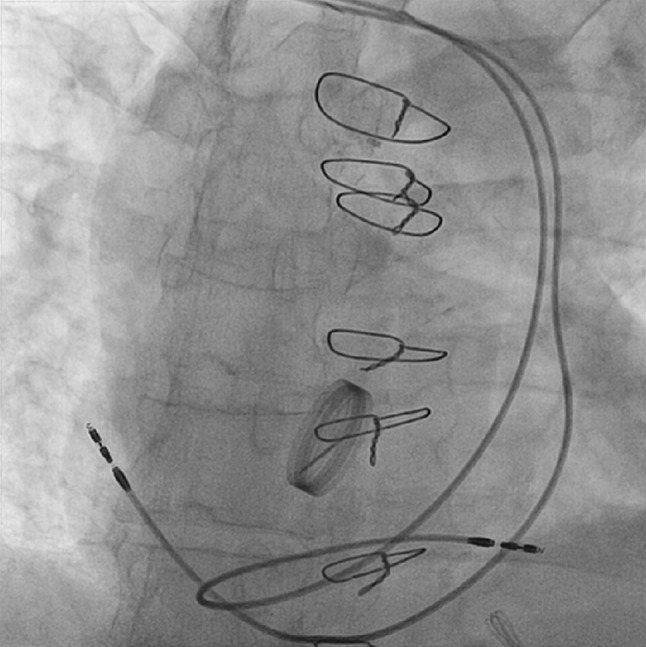
Radiograph showing final lead position in our patient

## Answer

You will find the answer elsewhere in this issue.

